# Cultural adaptation and validation of the desire to avoid pregnancy scale in Brazil

**DOI:** 10.1371/journal.pone.0327553

**Published:** 2025-07-28

**Authors:** Carolina Cavalcante da Silva Ale, Jennifer Hall, Geraldine Barrett, Corinne H. Rocca, Ana Luiza Vilela Borges

**Affiliations:** 1 Department of Public Health Nursing, School of Nursing, University of São Paulo, São Paulo, Brazil; 2 Institute for Women’s Health, University College London, London, United Kingdom; 3 Department of Obstetrics, Gynecology and Reproductive Sciences, Advancing New Standards in Reproductive Health (ANSIRH), School of Medicine, University of California, San Francisco, United States of America; UFSCar: Universidade Federal de Sao Carlos, BRAZIL

## Abstract

The development of valid measures of pregnancy intentions has been an important priority in the reproductive health field. A validated measure, the Desire to Avoid Pregnancy (DAP) scale, was developed in the USA to assess preferences regarding future pregnancy and childbearing, but it has not yet been validated in Brazil. This psychometric study aimed to adapt and evaluate the DAP scale in Brazilian Portuguese using both Item Response Theory and Classical Test Theory methods. Reproductive-aged women who had ever reported sexual activity, had not had a hysterectomy, were not sterilized, and had no partner with a vasectomy (n = 1,596) responded to an online survey with the 14 DAP scale items in March and April 2021. The items were comprehensible, even among women with lower education levels. Internal consistency (Cronbach’s α = 0.958) and test-retest reliability (ICC = 0.95) were both excellent. Exploratory factor analysis confirmed a one-factor model. Based on confirmatory factor analysis and the Item Response Model, items 3 (Thinking about becoming pregnant in the next 3 months makes me feel unhappy) and 5 (Becoming pregnant in the next 3 months would bring me closer to my main partner) did not perform well. However, testing a version without these items did not show substantial improvement in the psychometric parameters. The analysis showed significant differences in DAP scores according to age, educational status, and relationship status. As hypothesized, women with higher DAP scores were more likely to use contraception than those with lower scores [OR=2.12; 95%CI = 1.90–2.35]. The DAP scale, validated in Brazil, should be used in its full 14-item version until future studies are available. The scale has the potential to generate more accurate estimates of prospective pregnancy intention in Brazil and can be used to assess the outcomes of unintended pregnancies in maternal and infant health, providing an improvement over previous approaches.

## Introduction

Robust measurement of pregnancy intentions is a key component in the assessment of how sexual and reproductive health rights are safeguarded as well as whether progress towards some of the Sustainable Development Goals has been accomplished [1]. Existing methods of measuring pregnancy intention have shown that unintended pregnancies are still a public health concern [2], accounting for half of all pregnancies, many unsafe abortions, and maternal mortality, especially in low- and middle-income countries [3]. For some, unintended pregnancy reflects a lack of bodily and reproductive autonomy [4].

Many experts in the reproductive health field have pointed to an urgent need for improved approaches to measuring pregnancy intentions, desires and/or preferences. While the single question used in the Demographic and Health Surveys (DHS) (*“At the time you became pregnant with (name), did you want to become pregnant then, did you want to wait until later, or did you not want to have any (more) children at all?”*), for example, allows for comparison across more than 80 countries and over time, it is vulnerable to bias in the estimates of unintended births as there may be *ex post facto* rationalization [5], and the question cannot capture uncertainty, ambivalence or indifference about the pregnancy [6,7]. A retrospective measure that has been widely used is the London Measure of Unplanned Pregnancy (LMUP) [8]. Currently adapted for use in more than 20 countries, the LMUP is a six-item psychometrically validated scale that accounts for gradients of pregnancy intention, including ambivalence, and has been shown to be more reliable than the single DHS survey question [9].

However, it is only recently that a validated measure that allows the prospective assessment of preferences regarding a potential future pregnancy has been developed, the Desire to Avoid Pregnancy (DAP) scale. Based on a formative model, the DAP scale was developed in the United States of America (USA) [10], and adapted to other countries [11,12], like in the UK, where it was shown to be predictive of pregnancy, i.e., it was able to identify who was likely to become pregnant in a period of 12 months. This is of particular relevance as the DAP scale can contribute to clinical and research practices in terms of identifying individuals who either need contraception or preconception healthcare in addition to assessing the outcomes of pregnancies that take place irrespective of a strong desire to be avoided.

Like the LMUP, scores on the DAP scale fall on a continuous scale, enabling individuals’ perspectives on a possible future pregnancy to be measured in a more nuanced way, not limited to wanted/unwanted or intended/unintended categories. The DAP scale is comprised of 14 items that cover three conceptual domains (desires, feelings/attitudes, and practical consequences) referring to how women would feel about becoming pregnant in the next three months and having a baby in the next year. Response options are on a Likert scale, ranging from 0 to 4, from strongly disagree to strongly agree. The total score is an average, also ranging from 0 to 4: the higher the score, the higher the desire to avoid a pregnancy [10].

Both the DHS question and the LMUP scale have been used in Brazil in national and local studies; the former categorized almost half of births as unintended [13,14], while the latter found high levels of ambivalence among women who were currently pregnant or in the postpartum or post-abortion period [15]. However, the extent to which the DAP scale is reliable and valid among reproductive-aged women in Brazil is not known. This study aimed to adapt and evaluate the DAP scale in Brazil to be used among reproductive-aged women in order to assess pregnancy preferences in a prospective way and provide a more nuanced understanding of individuals’ reproductive preferences [16]. The availability of a valid psychometric instrument measuring pregnancy preferences can contribute to improved research on contraceptive needs and unintended pregnancies in Brazil to inform public health strategies and promote more person-centered reproductive healthcare. This work also contributes to global efforts to standardize and improve the measurement of pregnancy preferences across diverse populations.

## Materials and methods

### Study setting

This psychometric study was an online survey conducted in Brazil, Latin America.

### Translation process

In the cultural adaptation stage [17], two professional translators with no expertise in health science first translated the DAP scale items from English to Brazilian Portuguese. The two versions were compared by ALVB and CCSA (authors’ initials) and a third version created. Few words or terms needed adjustments. This version was analyzed by a group of five experts (four were experts in reproductive health and one in linguistics) who were aware of the objectives of the study and assessed if each of the translated items were equivalent to the original in four dimensions [18]: semantic, idiomatic, cultural and conceptual. Each expert completed an instrument where they could present their doubts on clarity of items and relevance and could suggest changes on the items, exclusion of words or even the whole item. Responses by the expert committee were summarized on a 4-point ordinal scale; they were asked to rate the items as not relevant (value = 1), somewhat relevant (value = 2), quite relevant (value = 3), and highly relevant (value = 4).

Informed by the experts’ feedback, the authors again revised the items and produced another version, which was back-translated to English and assessed by the authors of the original DAP scale. Through the notes and new adjustments, a revised version was produced and used during cognitive interviews. Cognitive interviews were conducted in November-December 2020 to assess women’s understanding of the DAP instructions, items, and response options. Interviewees aged 19–40 years were either invited by health community agents to be interviewed face-to-face in a primary health care facility in São Paulo city, or invited through the university social media for an online interview. Sample recruitment for cognitive interviews persisted until data saturation was achieved.

Beyond the DAP scale, we also prepared another structured instrument with questions about sociodemographic characteristics, reproductive history, contraceptive use and preconception measures for those who reported the intention to become pregnant. At the end, there was a question regarding their availability to complete the DAP scale again 15 days later. If they accepted, they provided their e-mail contact in order to be contacted again. Both DAP scale and sociodemographic questionnaire were administered via REDCap, an online survey software.

### Sample/eligibility criteria

Reproductive-aged women (18–49 years) who had ever reported sexual intercourse, had no history of hysterectomy, were not sterilized, nor had a partner who had undergone a vasectomy were eligible to take part in the study. An exclusion criterion was reporting a current pregnancy.

This psychometric study was based on a UK study which also aimed to evaluate the DAP, including its predictive ability, in a new context [11]. A sample size of 1000 reproductive aged women would be sufficient for psychometric studies, considering a minimum of 20 subjects per item [19] and to produce stable and accurate parameters for polytomous Item Response Theory (IRT) models under most data conditions [20], and was estimated to provide sufficient pregnancies to assess the predictive validity over 12 months follow up (not the subject of this paper).

From March 1 to April 30, 2021, the link to the DAP scale and the sociodemographic questionnaire was available on the University of São Paulo School of Nursing website and on social media (Instagram and Facebook). It was also sent via WhatsApp through networks and emailed through the mailing list of the institution. A specific website and hotline were created for this research. Social media boosts were implemented in the end of March 2021 in order to improve engagement and increase the number of women accessing the link. Recruitment should be closed when the target of 1,000 participants meeting eligiblity criteria and completing DAP scale was met. Respondents who completed the survey in the first two weeks were invited to take part in a test-retest phase. For those who were interested, a link to repeat the DAP instrument was sent to their e-mail 15 days later.

### Data analysis

We excluded every participant who did not meet inclusion criteria. Data analysis was conducted using both Classical Test Theory (CTT) and IRT, the latter to ensure comparability to the original study that developed the DAP scale. We examined the score distribution characteristics and the responses across each item, ensuring that no response category received more than 80% endorsement.

### Content validity

We obtained the content validity index (CVI) from the experts’ responses, which was calculated as the number of experts giving a rating 3 or 4 to the relevancy of each item, divided by the total number of experts, expressing the proportion of agreement on the relevancy of each item (the minimum acceptable CVI was 0.75) [21].

Cognitive interviews were conducted until data saturation was reached (n = 20). Women were purposefully selected to ensure a heterogeneous sample in terms of educational and social backgrounds, given that Brazil is among the countries with the highest levels of social inequality in the world [22]. Participants were first interviewed and then completed a semi-structured questionnaire, which allowed them to explain any difficulties in understanding and to suggest modifications to any part of the scale’s text. They also rated their comprehension of each item and of the overall scale (ranging from 0 to 100%). A comprehension score above 80% was considered acceptable [23]. Interviews were deliberately not audio-recorded to protect confidentiality and to promote participant comfort and spontaneity when discussing potentially stigmatizing content. All cognitive interviews were conducted by CCSA.

### CTT analysis

#### Structural validity.

We used factor analysis to assess structural validity. We conducted exploratory factor analysis (EFA) followed by confirmatory factor analysis (CFA) on the total sample. For the EFA, we used a polychoric correlation matrix appropriate for ordinal Likert-type data, with factor extraction via Unweighted Least Squares (ULS) and Robust Promin oblique rotation [24]. Retention criteria included parallel analysis with 500 simulations [25], Eigenvalue greater than 1, scree plot inspection, residual matrix analysis, communality greater than 0.40, factor loadings greater than 0.40, and total variance explained greater than 60% [26]. Sampling adequacy was verified using the Kaiser-Meyer-Olkin (KMO) test (with a value of more than 0.70 indicating suitability for factor analysis) [27]. The unidimensional solution was confirmed by Unidimensional Congruence (UniCo greater than 0.95), Explained Common Variance (ECV greater than 0.80), and Mean of Item Residual Absolute Loadings (MIREAL greater than 0.30) [28].

CFA was conducted using the Weighted Least Squares Mean and Variance adjusted (WLSMV) estimator, ideal for ordinal variables with a polychoric correlation structure [29]. Model fit was assessed using the Comparative Fit Index (CFI) (acceptable if ≥0.90), Goodness of Fit Index (GFI) (acceptable if ≥0.90), Mean Square Error of Approximation (RMSEA) (acceptable if less than 0.08), and Standardized Root Mean Square (SRMR) (acceptable if less than 0.08) [30].

### Internal consistency

Internal consistency was evaluated using Cronbach’s alpha, interpreted as acceptable equal or greater than 0.70; item-test correlation accepted if >0.50, and item-rest correlations if >0.20 [31,32].

### Test-retest reliability

It was expected that women’s desire to avoid pregnancy (measured construct) would be stable for the period between the baseline and 15 days later for the test-retest, as pregnancy intention may take more time to show considerable changes depending on personal contexts [33]; furthermore, it was shown to be stable over this time in the UK evaluation [11]. Women who answered the DAP scale at baseline and 15 days later were compared using McNemar test according to their sociodemographic characteristics. We assessed test-retest reliability with a two-way mixed effects intra-class coefficient (ICC) on women who completed a 2-week retest, looking for an ICC around 0.80 [34].

### Hypothesis testing for construct validity

Construct validity hypothesis testing was grounded in the literature and was used in an exploratory analysis of factors associated with the DAP score. Using multiple linear regression analysis, we hypothesized that older women, more educated women, women in a romantic relationship, and those who had been pregnant previously would have higher DAP scores. Construct validity was also tested using logistic regression analysis where we tested if women with a higher DAP score would be more likely to use contraception (dichotomous, considering all contraceptive methods in use at the moment of the interview, including withdrawal and fertility awareness), in the same way as performed in the original DAP psychometric analysis. Statistical significance was considered as p < 0.05.

### IRT analysis

IRT analysis consisted of fitting participant responses to a partial credit item response model (PCM). We assessed fit to the model using a weighted mean-squared fit (WMSF) t-statistic of 0.75–1.33 as a guideline of good fit [23]. In particular, we flagged values of >1.33 to indicate items that contribute least to the construct of pregnancy preferences [35]. Based on parameters from the PCM, we generated a Wright Map, which plots item (and item-threshold) locations adjacent to respondent locations; we examined the map to ensure that the DAP item-threshold locations appropriately covered the full range of women’s preference levels and that each item’s threshold locations were appropriately ordered. [35]. Finally, we assessed the monotonicity of each item (women’s responses to each item increased as their overall DAP scores increased) by examining the point biserial correlations.

Analyses were conducted in Stata V.17, Factor and ACER ConQuest V.5.9.0. The data used in this analysis are available in the University of São Paulo public scientific data repository (http://repositorio.uspdigital.usp.br/handle/item/767).

### Ethical statement

This study was approved by the University of São Paulo School of Nursing Research Ethics Committee in Brazil (number 4.252.351). Women invited to participate read the informed consent before deciding to accept or refuse participation. Only those who clicked on the “accept” button gained access to the instrument. We hosted a website and a hotline to clarify questions about the study as well as to provide information about sexual and reproductive health with a focus on family planning. Respondents did not receive any financial remuneration for participating in the study.

## Results

### Cultural adaptation and cognitive testing

After the translation from English to the Brazilian Portuguese, experts recommended adjustments in some expressions that would be more relevant and common to the context (e.g., enthusiastic rather than excited or thrilled) but showed more concern on item 5, which is about the partner (Becoming pregnant in the next 3 months would bring me closer to my main partner). They discussed whether there was a need to explain about the “romantic relationship” and concluded this would not be suitable for the Brazilian context. They also concluded that “main partner” would be more suitable than “the most important partner”, which was the translated term. Backtranslation to English indicated the version was idiomatic, cultural and conceptually equivalent to the original. The CVI was higher than 80% for all items.

The cognitive interviews confirmed that the DAP scale was easily understood by Brazilian women. A comprehension rate of 90% was recorded; the remaining 10% indicated discomfort with emotional terminology rather than a lack of understanding, highlighting the affective complexity surrounding reproductive intentions. There was no need to make any changes to syntax or wording. The Brazilian DAP used in the validation stage is presented in Supplemental File 1 (see Supporting Information in S1 File).

### Psychometric field test sample

**Fig 1:**
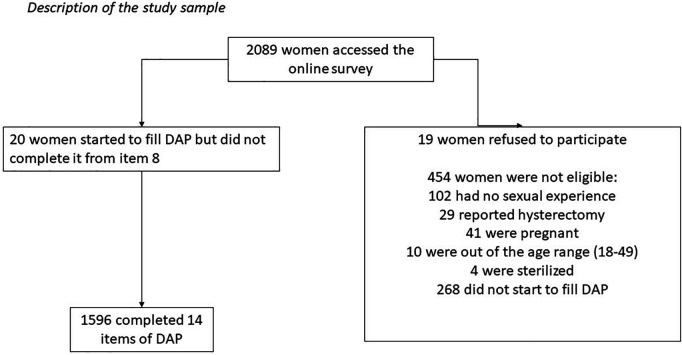
Description of study sample.

There were 2,089 accesses to the online survey. A total of 454 women were excluded from analyses due to being ineligible (Fig 1). Twenty women started to complete the DAP scale but did not continue (all of them interrupted from item 8), so they were also excluded. Our analysis consists of 1,596 women who completed all 14 items of the DAP scale.

Respondents were aged 18–49 years (mean = 29.0; SD = 7.1). The majority self-identified their skin color as white (64.7%), had a university degree (65.5%), had paid jobs (71.0%), lived in an urban area (97.0%), and were in a relationship (82.9%). Most had not been pregnant before (72.1%). Although a majority of our sample lived in the Southeast region (62.3%), the online survey reached women from all five regions of the country. Women who completed the DAP scale 15 days later for the retest (n = 158) were statistically different in terms of region of residence and history of pregnancy compared to women in the full sample (Table 1).

**Table 1:** Sociodemographic characteristics of participating women.

	Baseline		Followed 15 days later		*p* ^*^
Variables	*n*	*%*	*n*	*%*	
Age (years)					
18-24	524	32.8	62	39.2	0.175
25-34	713	44.7	66	41.7	
35-49	359	22.5	30	18.9	
Race/ethnicity					
White	1033	64.7	111	70.2	0.125
Black	510	32.0	47	29.8	
Other (Asian/ Indigenous)	53	3.3	–	–	
Education					
University degree	1045	65.5	101	63.9	0.666
No university degree	551	34.5	57	36.1	
Wealth Index					
High	324	20.3	32	20.2	0.799
Middle	927	58.1	95	60.1	
Low	345	21.6	31	19.6	
Religiosity					
No	387	26.9	50	31.6	0.205
Yes	1051	73.1	108	68.4	
Paid jobs					
No	464	29.0	46	29.1	0.990
Yes	1132	71.0	112	70.9	
Health insurance					
No	509	31.9	56	35.4	0.313
Yes	1087	68.1	102	64.6	
Region					
North	59	3.7	3	1.9	0.001
Northeast	198	12.4	11	6.9	
Middle-West	137	8.6	10	6.3	
Southeast	994	62.3	123	77.8	
South	208	13.0	11	7.0	
Relationship					
No	294	17.1	31	19.6	0.986
Yes, either married or not	1302	82.9	127	80.4	
Ever pregnant					
No	1151	72.1	128	81.0	0.008
Yes	445	27.9	30	19.0	
Total	1596	100.0	158	100.0	

* McNemar test.

### CTT analysis findings

**Fig 2:**
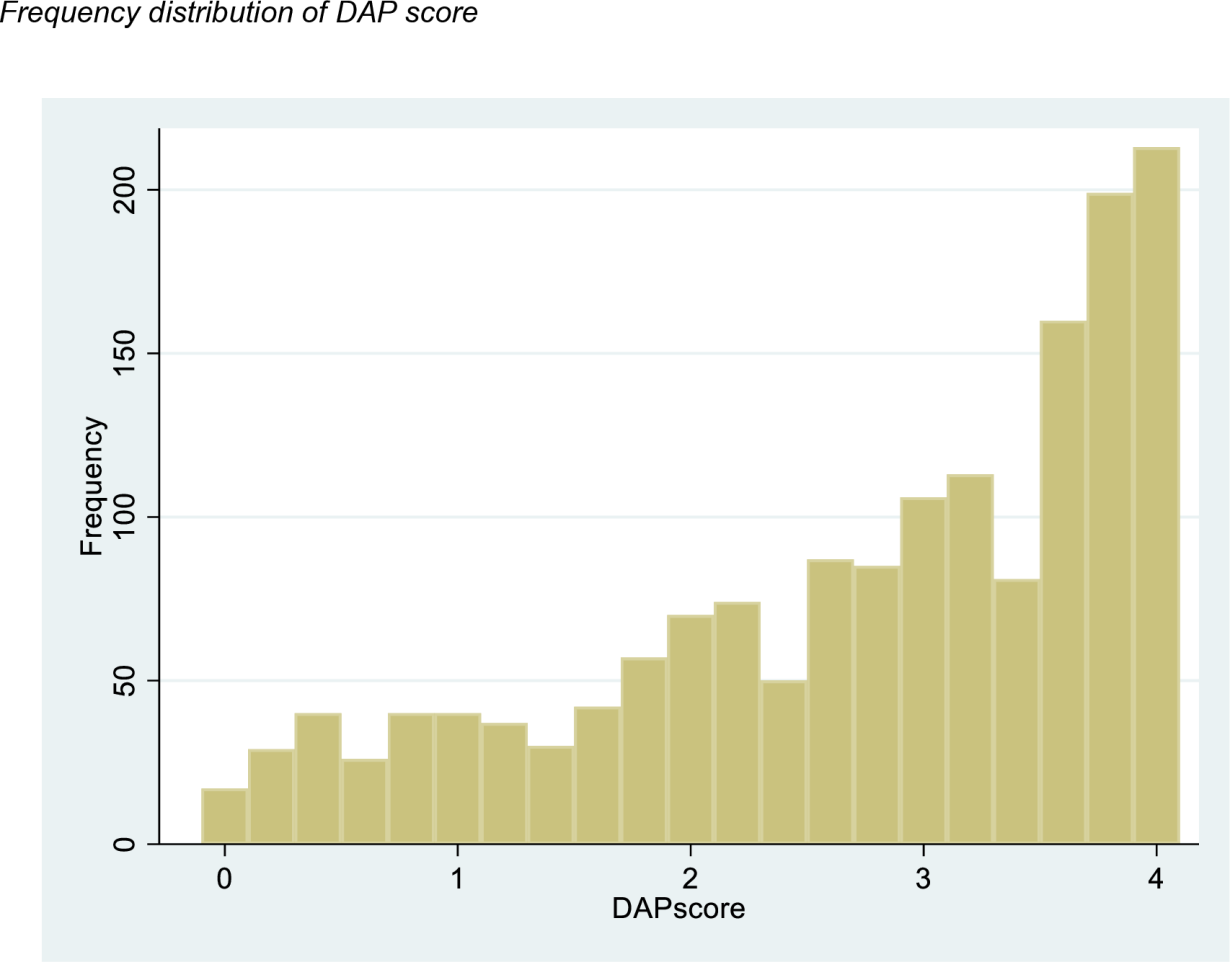
Frequency distribution of DAP score.

No item had an endorsement of more than 80%, but one of the categories (agree) of item 2 (It would be a good thing for me if I became pregnant in the next 3 months) had only 3.4% of the responses (see Supporting Information, Table in S2 File). DAP scores ranged from 0 to 4 (mean = 2.74; SD = 1.11) (see Supporting Information, Table in S3 File) and Fig 2 shows that they were left-skewed, with a higher proportion of women reporting higher DAP scores, i.e., a strong desire to avoid pregnancy.

EFA using principal factor extraction with no rotation conducted on the polychoric matrix (see Supporting Information, Table in S4 File) confirmed a one factor model with an Eigenvalue of 9.21 (LR test <0.001), explaining 76.2% of the variability of the responses (KMO = 0.97). The unidimensional solution was confirmed by UniCo (0.982), ECV (0.912), and MIREAL (0.218), supporting strong unidimensionality. All items loaded on to this one-factor and presented loadings above 0.40: 0.922 (item 1), 0.943 (item 2), 0.471 (item 3), 0.946 (item 4), 0.594 (item 5), 0.942 (item 6), 0.942 (item 7), 0.949 (item 8), 0.909 (item 9), 0.950 (item 10), 0.889 (item 11), 0.846 (item 12), 0.810 (item 13), and 0.869 (item 14). CFA on a one-factor model showed CFI = 0.997, GFI = 0.996, RMSEA = 0.064 and SRMR = 0.123.

Cronbach’s alpha was 0.958. All item-test correlations were positive (ranging from 0.446 on item 3 to 0.918 on item 10) and all item-rest correlations were also positive and greater than 0.35. Items 3 and 5 had the lowest item-test and item-rest correlations, and the scale’s internal consistency increased when they were excluded (Table 2).

**Table 2:** DAP scale score mean, standard deviation, item-test correlation, item-total correlation and reliability.

	Item	*M (SD)*	Item-test correlation	Item-rest correlation	Alpha without the item
1	I wouldn’t mind it if I became pregnant in the next 3 months.	3.06 (0.03)	0.859	0.833	0.956
2	It would be a good thing for me if I became pregnant in the next 3 months.	3.28 (0.03)	0.855	0.829	0.956
3	Thinking about becoming pregnant in the next 3 months makes me feel unhappy.	2.16 (0.04)	0.446	0.371	0.965
4	Thinking about becoming pregnant in the next 3 months makes me feel excited.	3.08 (0.03)	0.894	0.874	0.954
5	Becoming pregnant in the next 3 months would bring me closer to my main partner.	2.88 (0.03)	0.561	0.496	0.963
6	I want to have a baby within the next year.	3.02 (0.03)	0.889	0.869	0.955
7	If I had a baby in the next year, it would be bad for my life.	2.55 (0.04)	0.904	0.886	0.954
8	It would be a positive addition to my life to have a baby in the next year.	2.71 (0.03)	0.917	0.901	0.954
9	It would be the end of the world for me to have a baby in the next year.	1.96 (0.04)	0.832	0.801	0.956
10	Thinking about having a baby within the next year makes me smile.	2.63 (0.03)	0.918	0.903	0.954
11	Thinking about having a baby within the next year makes me feel stressed out.	2.73 (0.03)	0.856	0.829	0.956
12	I would feel a loss of freedom if I had a baby in the next year.	2.79 (0.03)	0.807	0.773	0.959
13	If I had a baby in the next year, it would be hard for me to manage raising the child.	2.54 (0.03)	0.779	0.741	0.957
14	I would worry that having a baby in the next year would make it harder for me to achieve other things in my life.	3.00 (0.03)	0.821	0.789	0.956

While total variability increased when items 3 and 5 were excluded, indicators for CFA remained quite stable (Table 3). Sensitivity analyses removing both items 3 and 5 improved scale reliability. Exclusion of the item 3 (Thinking about becoming pregnant in the next 3 months makes me feel unhappy) increased scale reliability (α = 0.97) but was retained due to acceptable correlations and factor loadings. Exclusion of the item 5 (Becoming pregnant in the next 3 months would bring me closer to my main partner) did not increase scale reliability (α = 0.96). CFA of the DAP without items 3 and 5 showed good model fit (CFI = 0.99, SRMR = 0.03), with an improvement in the total explained variance from 76.2 to 84.0% (Table 3).

**Table 3:** Indicators for EFA and CFA comparing the 14-item DAP scale with the exclusion of items 3 and 5.

Indicators		14-item DAP	DAP item 3 excluded	DAP item 5 excluded	DAP items 3 and 5 excluded
EFA	Parallel analysis (dimensions)	1	1	1	1
	Total explained variance (%)	76.2	80.3	79.5	84.0
	Cronbach’s alpha	0.96	0.97	0.96	0.98
	McDonald’s Omega	0.95	0.96	0.97	0.98
CFA					
	CFI	0.90	0.99	0.99	0.99
	GFI	0.90	0.99	0.99	0.99
	RMSEA	0.01	0.03	0.03	0.05
	SRMR	0.01	0.03	0.02	0.03

CFI = Comparative Fit Index, GFI = Goodness of Fit Index, RMSEA = Root Mean Square Error of Approximation, SRMR = Standardized Root Mean Square.

In terms of test-retest reliability, the ICC was 0.95 (95%CI 0.93–0.96), which indicates excellent stability over 15 days. The mean DAP score at baseline among the 158 women who completed the two-week retest was 2.83 (SD = 1.04) while the mean score of DAP among the whole sample was 2.70 (SD = 1.10).

The analysis of factors associated with DAP score using multiple linear regression showed a significant decrease in mean desire to avoid pregnancy with increasing age (β = −0.05 per year, 95%CI −0.06 to −0.04), higher education (β = −0.14, 95%CI −0.27 to −0.02) and being in a relationship (β = −0.43, 95%CI −0.56 to −0.31), but no significant difference by previous pregnancy. Logistic regression showed that women with higher DAP scores were more likely to use contraception than women with lower DAP scores (OR=2.12; CI = 1.91–2.36) (Table 4).

**Table 4:** Analysis of factors associated with DAP score.

Variables	Adjusted β^a^	95%CI		*p*
		*LL*	*UL*	
Age (in years)	−.05	−.06	−.04	
Education^c^	−.14	−.27	−.02	
In a relationship^d^	−.43	−.56	−.31	
Previous pregnancy^e^	−.11	−.24	.01	
	Odds Ratio^b^	95%CI		*p*
		*LL*	*UL*	
DAP score	2.12	1.91	2.35	

Note. total N = 1,596. CI = confidence interval; LL = lower limit; UL = upper limit.

^a^Multiple linear regression: DAP score.

^b^Logistic Regression: use of contraceptives (yes/no).

^c^0 = University degree, 1 = No university degree.

^d^0 = no, 1 = yes.

^e^0 = no, 1 = yes.

### IRT analysis findings

The IRT analysis showed that half of the items fit the PCM based on the WMSF (between 0.75 and 1.33), but eight items did not (see Supporting Information, Table in S5 File). Items 3 and 5 had relatively high fit statistics (2.83 and 2.13, respectively), which suggests frequent unexpected responses associated with these items. Items 8 and 10 had the lowest fit statistics (0.55), which suggests less variation than expected in participant responses. Centered item location estimates ranged from −0.70 logits for item 2, which was the easiest item for participants to endorse, to 1.05 logits for item 9, which was the most difficult item for participants to endorse.

Overall, the data suggests a nuanced spectrum of attitudes toward pregnancy among respondents. The Wright Map showed the full range of participant locations, with item thresholds falling across all levels of women’s preferences, indicating appropriate targeting. It also showed generalized item threshold locations were appropriately ordered (see Supporting Information, Fig in S6 File). Point-biserial correlations similarly were ordered as expected, indicating that women’s responses to each item increased as their overall DAP scores increased. The one exception is that for item 3, as the bottom three thresholds were misordered.

## Discussion

This study adapted the DAP scale to Brazilian Portuguese and assessed its validity using a comprehensive psychometric process, including forward and backward translation, expert committee and cognitive interviews. Findings indicated a high level of item understanding and acceptability as well as evidence of reliability according to accepted psychometric parameters.

The results showed that the Brazil DAP scale proved to be unidimensional as all items strongly loaded on to one factor. While this factor explained 76% of the variability of responses in Brazil, variance ranged from 57% in Turkey to 91% in the UK. Reliability, both in terms of internal consistency and test-retest, was good, just as in the USA, UK and Turkey [10–12].

Our sample in the Brazilian context using the DAP scale presented the highest mean score of evaluations to date. This difference may have occurred because Brazil was in the second COVID-19 lockdown during the interviews, which may have played an important role in pregnancy preferences during the pandemic just as observed elsewhere [36,37]. Both evaluations of the DAP scale in the UK and USA were done before the pandemic and in Turkey was performed predominantly among married women, so direct comparisons should be drawn with caution.

Factor analysis confirmed that the DAP scale adapted in Brazil is a unidimensional scale, which is slightly different from the analysis in the UK, where complementary analysis showed the need for further exploration of the factor structure in other samples [11]. All items showed factor loadings above the expected limit of 0.40, which confirms that the items contribute substantially to the measurement of the “desire to avoid pregnancy” construct represented by the factor [26].

While no item has shown to be below standard parameters in terms of reliability, items 3 and 5 showed correlation parameters far below the other items as well as the worst performance in the IRT PCM. Unlike in the USA and UK, item 3 was the second most difficult. Even women with high DAP scores overall responded frequently that they disagreed with this item. For the Brazilian culture, unhappiness (a term explicit in item 3) may sound aggressive when talking about a possible future pregnancy, especially considering that the other items refer to pregnancy in more nuanced and often positive ways. An expression of unhappiness when thinking of a future pregnancy may also relate to a non-desirable feeling that can lead to abortion, which is a very stigmatized issue in Brazil [38], since abortion faces many legal restrictions. Future use of the DAP scale in Brazil might test the use of a less aggressive word to capture negative feelings people might have in response to a pregnancy they did not intend, like “dissatisfied”, “unsatisfied” or even “sad”.

The relatively poor functioning of item 5 in the IRT analysis may reflect differences in wording and culture regarding partnership. The original DAP highlights that women need to consider the main partner and this may have confused women who were monogamous. Experts expressed that this was not a reasonable formulation for partnership but could not come to consensus on an alternative. Such discrepancies in the translation process were noted and resolved in a discussion between the authors, who chose to keep the same as the original. Literature highlights the importance of the partner in defining pregnancy preferences [39,40] and we still do not know to what extent they influence future intentions towards a potential pregnancy. Another concern is that item 5 also seemed to be problematic in the USA and UK validations, which is an issue that needs to be considered in future studies.

While some of the parameters regarding these items may fall below acceptable standards, the assessment of an abbreviated measure did not dramatically improve the overall psychometric quality of the Brazil DAP scale. The decision not to exclude items 3 and 5 based only on IRT results is based on the fact that most CTT parameters, like reliability, were good [35]. Additionally, few studies on the validation of DAP are available so far and changes of such magnitude may result in a (somewhat) different scale compared to the original validated in the USA and other countries, which may not be an advantage [41]; their exclusion would prevent full comparison between the DAP scale available in Brazil and in those countries.

IRT analysis showed that item 2 was the easiest item for participants to endorse (It would be a good thing for me if I became pregnant in the next 3 months). Similarly, this item presented the best performance for predicting a chance of pregnancy within 12 months among UK women who reported they strongly agreed, indicating that the use of a single-item DAP in clinical setting may be viable [42]. Future studies on this topic are necessary to refine and assess DAP scale properties. For now, the DAP scale validated in Brazilian Portuguese is to be used in its full 14-item version in Brazil until future studies are conducted, including an assessment of a short-version and a single-item DAP performance.

A prospective measure like the DAP scale is valuable and useful for improving policy and programmatic decisions, monitoring women who need either preconception or contraception care and assessing maternal and infant outcomes from unintended pregnancies [16]. The DAP scale would also be useful for screening adolescents classified with a low desire to avoid pregnancy. For the Brazilian context, with high adolescent fertility rates [43], this would be a useful instrument to anticipate those who would be willing to become pregnant and then tailor interventions to respond to their sexual and reproductive health needs. However, we have not tested the scale among adolescents, therefore there is a gap in our knowledge about its performance in this group.

Another limitation is that this is a highly educated sample of women, far more educated than Brazilian women in general [44]. The reason for such discrepancy may be due to our approach of inviting participants from the university contacts and network. While we interviewed women from a wide range of educational backgrounds, the sample shows some failure to reach the most disadvantaged populations, like the non-white and less educated groups, who have more difficulties in accessing surveys online [45] and therefore were underrepresented.

## Conclusion

The Brazilian DAP scale is a valid and reliable unidimensional measurement of pregnancy preferences. The scale was easily understood by women, including less educated women. While IRT analyses raised concerns about items 3 and 5 – concerning unhappiness towards a future pregnancy and the role of the partner, respectively – a version of the scale without them was not substantially better. This suggests that the 14-item scale can be used in the Brazilian context in clinical settings and epidemiological studies, since it presents the potential to generate more accurate estimates of prospective pregnancy intention and its relationship with maternal and infant health compared to previous measures, and the opportunity to make international comparisons.

## Supporting information

S1 File Desire to Avoid Pregnancy (DAP), Brazilian version – final version in Brazilian Portuguese. (DOCX)

S2 File Table – DAP responses according to each item. (DOCX)

S3 File Table – DAP score means and standard deviations according to sociodemographic characteristics. (DOCX)

S4 File Table – Polychoric correlation matrix. (DOCX)

S5 File Table – DAP scale items by domain with item fit and location estimate. (DOCX)

S6 File Fig – Wright Map. (TIF)
