# Supplementary material for: Cultural adaptation and validation of the desire to avoid pregnancy scale in Brazil
Source: PLoS One. 2025 Jul 28;20(7):e0327553. doi: 10.1371/journal.pone.0327553 (PMC12303264; doi:10.1371/journal.pone.0327553)
Supplement: S1 File — (DOCX) [file pone.0327553.s001.docx]

**Supplementary File 1**

Escala *Desire to Avoid Pregnancy* (DAP) – Brasil

| As perguntas a seguir são sobre seus pensamentos e sentimentos atuais com relação à ideia de engravidar nos próximos três meses e de ter um bebê no próximo ano.  Sabemos que as mulheres têm pensamentos e sentimentos muito diferentes sobre uma gravidez e sobre ter um bebê, e que eles podem mudar ao longo do tempo.  Lembre-se de que não há respostas certas ou erradas  (Escala Likert de 5 pontos: Concordo totalmente; Concordo; Não concordo, nem discordo; Discordo; Discordo totalmente)  1. Eu não me importaria se eu engravidasse nos próximos três meses.  2. Seria bom para mim se eu engravidasse nos próximos três meses.  3. Pensar em engravidar nos próximos três meses faz com que eu me sinta infeliz.  4. Pensar em engravidar nos próximos três meses faz com que eu me sinta empolgada  5. Engravidar nos próximos três meses me aproximaria do meu parceiro mais importante (por parceiro mais importante, nós queremos dizer aquele que você leva mais a sério.  Se você não tem um parceiro romântico, pense na pessoa com que você teve relações sexuais mais recentemente).  As afirmativas a seguir são sobre seus pensamentos e sentimentos com relação à ideia de ter um BEBÊ NO PRÓXIMO ANO.  Mesmo se achar que não pode ter um bebê, imagine em como você se sentiria sobre TER UM BEBÊ.  6. Eu quero ter um bebê no próximo ano.  7. Se eu tivesse um bebê no próximo ano, seria ruim para minha vida.  8. Ter um bebê no próximo ano seria um ganho para minha vida.  9. Ter um bebê no próximo ano seria o fim do mundo para mim.  10. Pensar em ter um bebê no próximo ano me faz sorrir.  11. Pensar em ter um bebê no próximo ano me deixa estressada.  12. Eu sentiria a perda da minha liberdade se tivesse um bebê no próximo ano.  13. Se eu tivesse um bebê no próximo ano, seria difícil para mim conseguir criar a criança.  14. Eu me preocuparia em ter um bebê no próximo ano pois tornaria mais difícil para mim alcançar outras coisas na minha vida. |
| --- |
