# Supplementary material for: Cultural adaptation and validation of the desire to avoid pregnancy scale in Brazil
Source: PLoS One. 2025 Jul 28;20(7):e0327553. doi: 10.1371/journal.pone.0327553 (PMC12303264; doi:10.1371/journal.pone.0327553)
Supplement: S2 File — (DOCX) [file pone.0327553.s002.docx]

**Supplementary File 2**

**Table**

*DAP responses according to each item*

|  | **Item** | **Strongly Agree** | | **Agree** | | **Neither Agree Nor Disagree** | | **Disagree** | | **Strongly Disagree** | |
| --- | --- | --- | --- | --- | --- | --- | --- | --- | --- | --- | --- |
|  |  | **n** | **%** | **n** | **%** | **n** | **%** | **n** | **%** | **n** | **%** |
| 1 | I wouldn’t mind it if I became pregnant in the next 3 months. | 175 | 11.0 | 105 | 6.6 | 127 | 8.0 | 235 | 14.7 | 954 | 59.8 |
| 2 | It would be a good thing for me if I became pregnant in the next 3 months. | 126 | 7.9 | 55 | 3.4 | 116 | 7.3 | 249 | 15.6 | 1050 | 65.8 |
| 3 | Thinking about becoming pregnant in the next 3 months makes me feel unhappy. | 520 | 32.6 | 222 | 13.9 | 244 | 15.3 | 209 | 13.1 | 401 | 25.1 |
| 4 | Thinking about becoming pregnant in the next 3 months makes me feel excited. | 127 | 8.0 | 105 | 6.6 | 189 | 11.8 | 260 | 16.3 | 915 | 57.3 |
| 5 | Becoming pregnant in the next 3 months would bring me closer to my main partner. | 115 | 7.2 | 178 | 11.2 | 284 | 17.8 | 226 | 14.2 | 793 | 49.7 |
| 6 | I want to have a baby within the next year. | 157 | 9.8 | 125 | 7.8 | 144 | 9.0 | 264 | 16.5 | 906 | 56.8 |
| 7 | If I had a baby in the next year, it would be bad for my life. | 617 | 38.7 | 262 | 16.4 | 298 | 18.7 | 226 | 14.2 | 193 | 12.1 |
| 8 | It would be a positive addition to my life to have a baby in the next year. | 146 | 9.1 | 168 | 10.5 | 335 | 21.0 | 298 | 18.7 | 649 | 40.7 |
| 9 | It would be the end of the world for me to have a baby in the next year | 381 | 23.9 | 222 | 13.9 | 254 | 15.9 | 330 | 20.7 | 381 | 23.9 |
| 10 | Thinking about having a baby within the next year makes me smile. | 177 | 11.1 | 194 | 12.2 | 284 | 17.8 | 328 | 20.6 | 613 | 38.4 |
| 11 | Thinking about having a baby within the next year makes me feel makes me feel stressed out. | 637 | 39.9 | 390 | 24.4 | 219 | 13.7 | 205 | 12.8 | 145 | 9.1 |
| 12 | I would feel a loss of freedom if I had a baby in the next year. | 685 | 42.9 | 374 | 23.4 | 206 | 12.9 | 186 | 11.7 | 145 | 9.1 |
| 13 | If I had a baby in the next year, it would be hard for me to manage raising the child. | 563 | 35.3 | 358 | 22.4 | 222 | 13.9 | 293 | 18.4 | 160 | 10.0 |
| 14 | I would worry that having a baby in the next year would make it harder for me to achieve other things in my life. | 830 | 52.0 | 341 | 21.4 | 144 | 9.0 | 163 | 10.2 | 118 | 7.4 |
