# Supplementary material for: Cultural adaptation and validation of the desire to avoid pregnancy scale in Brazil
Source: PLoS One. 2025 Jul 28;20(7):e0327553. doi: 10.1371/journal.pone.0327553 (PMC12303264; doi:10.1371/journal.pone.0327553)
Supplement: S3 File — (DOCX) [file pone.0327553.s003.docx]

**Supplementary File 3**

**Table**

*DAP score means and standard deviations according to sociodemographic characteristics*

| **Variables** | ***M*** | ***SD*** |
| --- | --- | --- |
| **Age (years)** |  |  |
| 18-24 | 3.33 | 0.75 |
| 25-34 | 2.59 | 1.09 |
| 35-49 | 2.18 | 1.16 |
| **Race/ethnicity** |  |  |
| White | 2.80 | 1.06 |
| Black | 2.66 | 1.16 |
| Other (Asian/ Indigenous) | 2.45 | 1.21 |
| **Education** |  |  |
| University degree | 2.53 | 1.11 |
| No university degree | 3.13 | 0.98 |
| **Wealth Index** |  |  |
| High | 2.83 | 1.08 |
| Middle | 2.64 | 1.11 |
| Low | 2.93 | 1.08 |
| **Religion** |  |  |
| None | 3.09 | 0.98 |
| Catholic | 2.44 | 1.09 |
| Protestant | 2.39 | 1.15 |
| Others^‖^ | 2.65 | 1.12 |
| **Paid jobs** |  |  |
| No | 3.03 | 0.99 |
| Yes | 2.62 | 1.12 |
| **Health insurance** |  |  |
| No | 2.86 | 1.07 |
| Yes | 2.68 | 1.11 |
| **Region** |  |  |
| North | 2.61 | 1.18 |
| Northeast | 2.64 | 1.23 |
| Middle-West | 2.64 | 1.12 |
| Southeast | 2.78 | 1.07 |
| South | 2.74 | 1.09 |
| **Romantic relationship** |  |  |
| No | 3.23 | 0.83 |
| Yes | 2.63 | 1.12 |
| **Ever pregnant** |  |  |
| No | 2.90 | 1.06 |
| Yes | 2.31 | 1.10 |
| **Total** | 2.74 | 1.10 |
|  |  |  |
