# Supplementary material for: Cultural adaptation and validation of the desire to avoid pregnancy scale in Brazil
Source: PLoS One. 2025 Jul 28;20(7):e0327553. doi: 10.1371/journal.pone.0327553 (PMC12303264; doi:10.1371/journal.pone.0327553)
Supplement: S4 File — (DOCX) [file pone.0327553.s004.docx]

**Supplementary File 4**

**Table**

*Polychoric correlation matrix*

|  | DAP 1 | DAP 2 | DAP 3 | DAP 4 | DAP 5 | DAP 6 | DAP 7 | DAP 8 | DAP 9 | DAP 10 | DAP 11 | DAP 12 | DAP 13 | DAP 14 |
| --- | --- | --- | --- | --- | --- | --- | --- | --- | --- | --- | --- | --- | --- | --- |
| DAP 1 | 1.0 | - | - | - | - | - | - | - | - | - | - | - | - | - |
| DAP 2 | 0.9 | 1.0 | - | - | - | - | - | - | - | - | - | - | - | - |
| DAP 3 | 0.4 | 0.4 | 1.0 | - | - | - | - | - | - | - | - | - | - | - |
| DAP 4 | 0.9 | 1.0 | 0.4 | 1.0 | - | - | - | - | - | - | - | - | - | - |
| DAP 5 | 0.6 | 0.6 | **0.1** | 0.7 | 1.0 | - | - | - | - | - | - | - | - | - |
| DAP 6 | 0.9 | 0.9 | 0.4 | 0.9 | 0.6 | 1.0 | - | - | - | - | - | - | - | - |
| DAP 7 | 0.8 | 0.9 | 0.5 | 0.9 | 0.5 | 0.9 | 1.0 | - | - | - | - | - | - | - |
| DAP 8 | 0.9 | 0.9 | 0.5 | 0.9 | 0.6 | 0.9 | 0.9 | 1.0 | - | - | - | - | - | - |
| DAP 9 | 0.8 | 0.8 | 0.5 | 0.8 | 0.5 | 0.8 | 0.9 | 0.9 | 1.0 | - | - | - | - | - |
| DAP 10 | 0.9 | 0.9 | 0.5 | 0.9 | 0.6 | 0.9 | 0.9 | 0.9 | 0.9 | 1.0 | - | - | - | - |
| DAP 11 | 0.8 | 0.8 | 0.5 | 0.8 | 0.5 | 0.8 | 0.9 | 0.8 | 0.9 | 0.8 | 1.0 | - | - | - |
| DAP 12 | 0.7 | 0.8 | 0.4 | 0.8 | 0.4 | 0.8 | 0.8 | 0.8 | 0.8 | 0.8 | 0.8 | 1.0 | - | - |
| DAP 13 | 0.7 | 0.7 | 0.4 | 0.7 | 0.4 | 0.7 | 0.8 | 0.8 | 0.8 | 0.7 | 0.7 | 0.7 | 1.0 | - |
| DAP 14 | 0.8 | 0.8 | 0.4 | 0.8 | 0.4 | 0.8 | 0.8 | 0.8 | 0.8 | 0.8 | 0.8 | 0.8 | 0.8 | 1.0 |
