# Supplementary material for: Cultural adaptation and validation of the desire to avoid pregnancy scale in Brazil
Source: PLoS One. 2025 Jul 28;20(7):e0327553. doi: 10.1371/journal.pone.0327553 (PMC12303264; doi:10.1371/journal.pone.0327553)
Supplement: S5 File — (DOCX) [file pone.0327553.s005.docx]

**Supplementary File 5**

**Table**

*DAP scale items by domain with item fit and location estimate*

|  | **Item** | **Item fit** | **Item location** |
| --- | --- | --- | --- |
|  | **Cognitive Desires and Preferences** | **Weighted MNSQ** | **Brazil**  **estimate** |
| 1 | I wouldn’t mind it if I became pregnant in the next 3 months | 0.81 | -0.33 |
| 2 | It would be a good thing for me if I became pregnant in the next 3 months | 0.66 | -0.70 |
| 6 | I want to have a baby within the next year | 0.64 | -0.32 |
| 7 | If I had a baby in the next year, it would be bad for my life | 0.58 | 0.25 |
| 8 | It would be a positive addition to my life to have a baby in the next year | 0.55 | 0.01 |
| 9 | It would be the end of the world for me to have a baby in the next year | 0.77 | 1.05 |
|  | **Affective Feelings and Attitudes** |  |  |
| 3 | Thinking about becoming pregnant in the next 3 months makes me feel unhappy | 2.83 | 0.84 |
| 4 | Thinking about becoming pregnant in the next 3 months makes me feel excited | 0.59 | -0.46 |
| 10 | Thinking about having a baby within the next year makes me smile | 0.55 | 0.16 |
| 11 | Thinking about having a baby within the next year makes me feel makes me feel stressed out | 0.83 | -0.01 |
|  | **Anticipated Practical Consequences** |  |  |
| 5 | Becoming pregnant in the next 3 months would bring me closer to my main partner | 2.13 | -0.26 |
| 12 | I would feel a loss of freedom if I had a baby in the next year | 1.05 | -0.08 |
| 13 | If I had a baby in the next year, it would be hard for me to manage raising the child | 1.19 | 0.28 |
| 14 | I would worry that having a baby in the next year would make it harder for me to achieve other things in my life | 0.97 | -0.38 |
