# Supplementary figures and images for: Cultural adaptation and validation of the desire to avoid pregnancy scale in Brazil
Source: PLoS One. 2025 Jul 28;20(7):e0327553. doi: 10.1371/journal.pone.0327553 (PMC12303264; doi:10.1371/journal.pone.0327553)

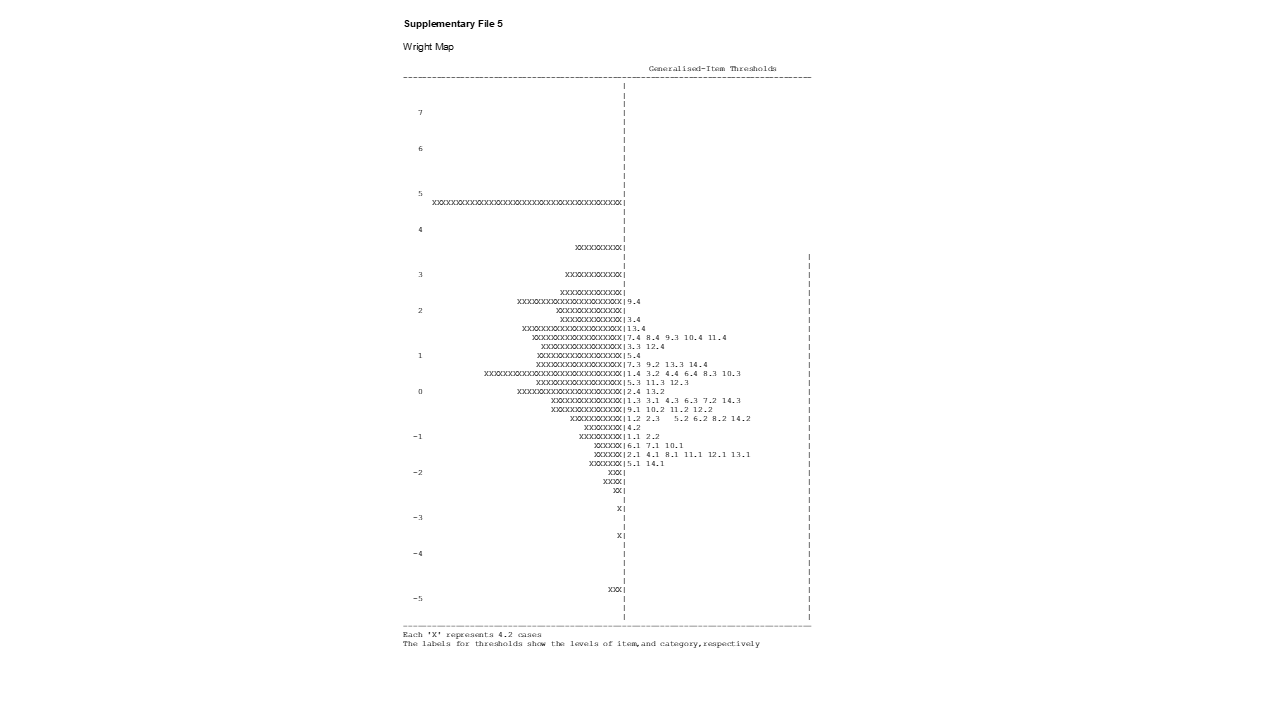

Supplement: S6 File — (TIF) [file pone.0327553.s006.tif]
